# Pregabalin for postherpetic itch: a case report

**DOI:** 10.1186/s40981-020-00330-x

**Published:** 2020-03-23

**Authors:** Nobuhiro Shimada, Yasunori Niwa, Kunihisa Hotta, Takashi Igarashi, Mamoru Takeuchi

**Affiliations:** Department of Anesthesiology and Critical Care Medicine, Jichi Medical University, 3311-1, Yakushiji, Shimotsuke, Tochigi, 329-0498 Japan

**Keywords:** Postherpetic itch, Postherpetic pruritus, Neuropathic itch, Pregabalin

## Abstract

**Background:**

Postherpetic itch has not commonly received attention as a complication of herpes zoster because pain predominates over itch in most patients with herpes zoster. Most cases of postherpetic itch are mild; however, cases of severe postherpetic itch reducing quality of life are rare.

**Case presentation:**

A 52-year-old woman complained of severe itch in her left pinna and cheek 1 month after the first onset of herpes zoster at the same site. Owing to her scratching, she developed ulcers on her left pinna and cheek. Pregabalin was prescribed, and the itch subsided immediately, with the ulcers disappearing within 1 month.

**Discussion:**

Severe itch was thought to be caused by neural injury from herpes zoster. Pregabalin may be a useful treatment option for neuropathic itch induced by herpes zoster.

## Background

Postherpetic itch (PHI), one of the complications of herpes zoster, may reduce quality of life of patients, similar to that observed in postherpetic neuralgia (PHN) [1]. However, PHI is not commonly recognized as a complication of herpes zoster because pain predominates over itch for most patients [2–4]. Therefore, only few studies have reported the epidemiology and clinical characteristics of PHI [2–4], and a standard treatment for PHI has not been established to date, although various treatments for PHI have been attempted [5–9]. In our patient, severe PHI subsided after treatment with pregabalin.

## Case presentation

**Fig. 1:**
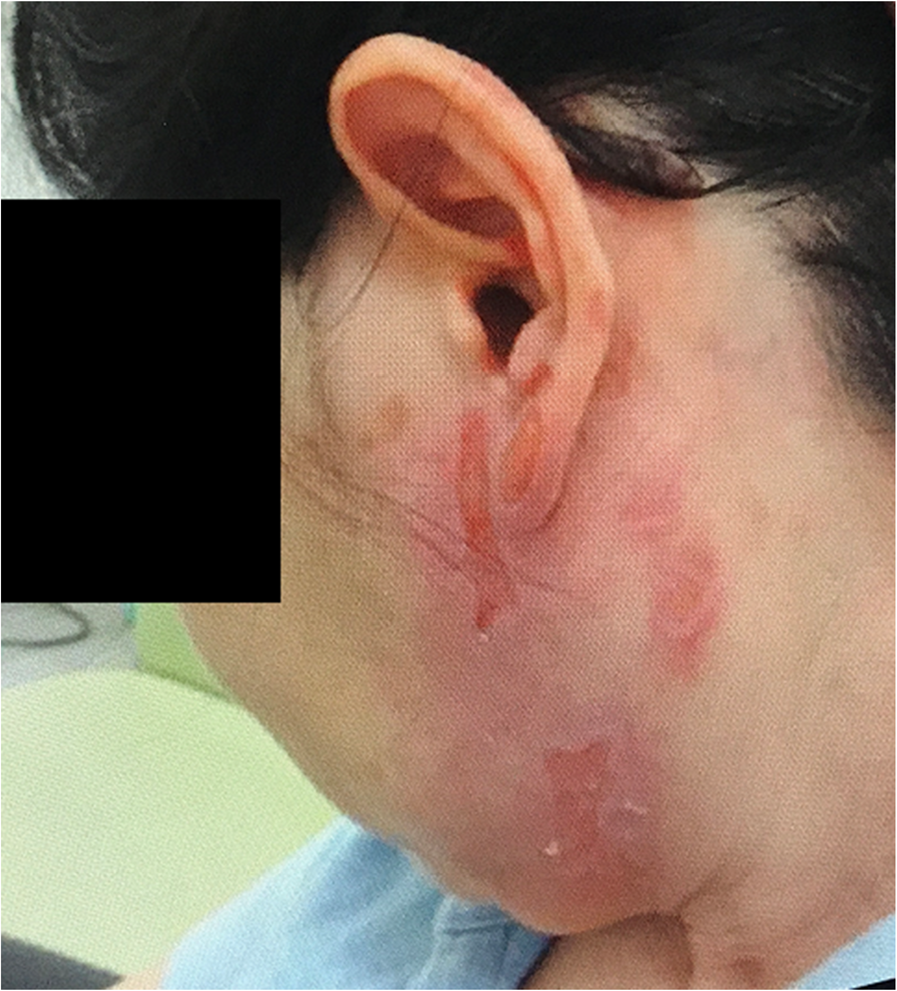
Cutaneous findings of the left pinna and cheek. The rash of herpes zoster had improved and only pigmentation was remained. Ulcers due to scratching were observed

A 52-year-old woman presented at our institution with herpes zoster. She had a medical history of myelodysplastic syndromes, cerebral infarction, and dysthymia treated with fluvoxamine. The aggregated rash with erythema and blisters accompanied by shooting pain and allodynia of the left pinna and cheek was typical of herpes zoster affecting the cervical dermatomes C2 and C3. The patient was treated with acyclovir (4000 mg/day) and acetaminophen (2000 mg/day). The rash improved and the pain completely disappeared within 1 month of this treatment. At 1 month after the first onset of the rash, itch appeared in the same dermatomal distribution involving the left pinna and cheek. The itch became severe, and the patient scratched her left pinna and cheek all day. The severity of the itch according to a 10-point numerical rating scale (NRS) was 8 during the day, with occasional exacerbations to 10 at night. The patient complained of sleeplessness, with severe itch that was evoked by the light touch of bedclothes. The patient was referred to our department 1 month after the first onset of the itch. On examination, blisters of the left pinna and cheek had already disappeared, leaving pigmentation only, and ulcers due to scratching were observed (Fig. 1). There was a decrease in skin perception with touching, warmth, and cold as well as painful stimuli in her left pinna and cheek. Alloknesis (characterized by itch caused by innocuous mechanical stimulation) was identified in the same site. There was neither spontaneous pain nor allodynia after the rash had disappeared. Furthermore, the Hospital Anxiety and Depression Scale was 5 points for anxiety and 4 points for depression. Oral use of levocetirizine and diphenhydramine ointment did not relieve the itch. Pregabalin (25 mg) was taken once a day regularly after obtaining informed consent for the off-label use of pregabalin. Ten days later, the severity of the itch according to NRS had improved to 3. The patient was able to sleep without exacerbation of the itch at night. Her ulcers from scratching improved within 1 month of treatment with pregabalin. Given that 50 mg/day of pregabalin induced daytime sleepiness, the patient was maintained at 25 mg/day. One month after initiating pregabalin, the severity of the itch according to NRS had improved to 1, only slight pigmentation due to the herpes zoster remained, and no ulcers were observed. The patient was maintained at 25 mg/day of pregabalin and eventually returned to her usual life with slight concern for the itch.

## Discussion

In herpes zoster, 29%–46% of patients in the acute phase and 38%–62% of patients in the chronic phase develop itch [2–4]. However, pain is the main symptom rather than itch in 88% of patients, and pain reduces quality of life [2–4]. Therefore, itch is often left unattended in such cases, whereas pain is frequently treated. Conversely, severe cases wherein itch was the main symptom and threatened quality of life have been rarely reported [1]. The rarity of our case was that the patient had no pain but had itching, which was severe enough to cause ulcers.

The pathophysiology of chronic pruritus can be classified under neurogenic, psychogenic, or neuropathic etiologies [10]. In this case, we diagnosed the itch as neuropathic based on the findings of alloknesis, which appeared in the same distribution of hypoesthesia confined to two adjacent dermatomes. We considered the likely involvement of neurogenic itch to be low because the rash had disappeared. Furthermore, while the patient had a medical history of dysthymia, psychogenic itch was unlikely based on the findings of the Hospital Anxiety and Depression Scale.

There are two mechanisms of itching in herpes zoster depending on the time course. The first mechanism is a neurogenic itch mediated due to histamine in the acute phase when the rash appears [11]. The second mechanism of itching is neuropathic itch or PHI caused by neural injury in the chronic phase of herpes zoster after the rash has disappeared [10, 12]. In neuropathic itch, varicella-zoster virus alleviates and demyelination of nerve fibers in the skin that transmit itch occurs [1], which is similar to neuropathic pain [13, 14]. Demyelination of nerve fibers could cause ectopic discharges due to changes in ion channels [15, 16], and overexcitation of primary neurons increases the transmission of itch, resulting in intractable neuropathic itch.

Our patient had no pain and only neuropathic itch. We believe that itch without pain may promote more severe symptoms than itch with pain. Skin damage due to scratching normally induces the pain, which serves as a protective mechanism against scratching. However, when itch occurs in painless areas, these protective mechanisms would not work. As a result, a patient who has painless itch would continue to scratch despite the resulting skin damage becoming severe [1]. Therefore, neuropathic itch in painless areas may lead to dangerous symptoms.

In theory, antihistamines are not effective for such neuropathic itch [1, 6, 8], as observed in our patient. The treatment for intractable neuropathic itch is difficult, and several treatments have been attempted, including gabapentin [5], carbamazepine [6], nerve block [7], stellate ganglion block [8], and pulsed radiofrequency [9]. Nalfurafine, which serves as a kappa opioid agonist, may also be effective for the treatment of PHI. We believe that several drugs that alleviate neuropathic pain are effective for relief of PHI, given the mechanism of neuropathic itch noted above [1, 10, 12]. We selected pregabalin for our patient. Although serotonin norepinephrine reuptake inhibitors and tricyclic antidepressants were other options, their use in our patient was difficult because she was already using a selective serotonin reuptake inhibitor. Pregabalin is a γ-amino acid analog that binds to the α_2_δ-subunit of voltage-dependent calcium channels in the dorsal root ganglion, suppressing the release of neurotransmitters and thus relieves neuropathic pain [17]. Taken together, we infer that pregabalin suppresses neuropathic itch in PHI with similar mechanisms in neuropathic pain [17].

Many studies on chronic pruritus have reported that pregabalin is effective at a dose of 75 mg/day or more [18]. In the present case, we started pregabalin at a small dose of 25 mg/day to prevent side effects, such as dizziness. This low dose was satisfactory for this patient, despite another study reporting a case in which 150 mg/day of pregabalin was ineffective for PHI treatment [7]. The reason for the effectiveness of a small amount of pregabalin in this case could be that the patient had a poor physique with poor general condition after cerebral infarction. Pregabalin was effective for our PHI patient, and could therefore also be a useful treatment for other PHI cases.

## Data Availability

All data generated or analyzed during this study are included in this published article.

## Abbreviations

PHI: Postherpetic itch
PHN: Postherpetic neuralgia
NRS: Numerical rating scale
